# The Effect of Intensive Dietary Intervention on the Level of RANTES and CXCL4 Chemokines in Patients with Non-Obstructive Coronary Artery Disease: A Randomised Study

**DOI:** 10.3390/biology10020156

**Published:** 2021-02-16

**Authors:** Magdalena Makarewicz-Wujec, Jan Henzel, Mariusz Kruk, Cezary Kępka, Łukasz Wardziak, Piotr Trochimiuk, Andrzej Parzonko, Marcin Demkow, Zofia Dzielińska, Malgorzata Kozłowska-Wojciechowska

**Affiliations:** 1 Department of Clinical Pharmacy and Pharmaceutical Care, Medical University of Warsaw, 02-091 Warsaw, Poland; mkw@wum.edu.pl; 2 Department of Coronary and Structural Heart Diseases, Cardinal Stefan Wyszynski Institute of Cardiology, 04-628 Warsaw, Poland; jhenzel@ikard.pl (J.H.); mkruk@ikard.pl (M.K.); ckepka@ikard.pl (C.K.); lukas.wardziak@gmail.com (Ł.W.); ptrochimiuk@ikard.pl (P.T.); mdemkow@ikard.pl (M.D.); zdzielinska@ikard.pl (Z.D.); 3 Department of Pharmacognosy and Molecular Basis of Phytotherapy, Medical University of Warsaw, 02-091 Warsaw, Poland; andrzej.parzonko@wum.edu.pl

**Keywords:** DASH diet, chemokine CXCL4, chemokine RANTES, atherosclerosis, inflammation

## Abstract

**Simple Summary:**

The dietary approaches to stop hypertension (DASH) diet contains meals with plenty of vegetables, fruits and low-fat dairy products, as well as whole grains, fish, poultry and nuts. One of the potential mechanisms of the beneficial effects of the DASH diet on the cardiovascular system may be modification of the inflammatory process. In the present study, we assessed the impact of the DASH diet on selected inflammatory markers in patients with atherosclerosis. The study lasted 12 months and involved 79 patients (40 followed the DASH diet, while 39 were in the control group). The results indicate that the DASH diet lowers the level of the inflammatory markers, which may contribute to the inhibition of atherosclerosis.

**Abstract:**

Background: Inflammation is the key pathophysiological mechanism of the initiation and progression of atherosclerosis. The study objective was to assess the effects of a dietary intervention based on the model of the dietary approaches to stop hypertension (DASH) diet on the levels of chemokines RANTES and CXCL4 in patients with non-obstructive coronary artery disease. Methods: As part of Dietary Intervention to Stop Coronary Atherosclerosis in Computed Tomography (DISCO-CT) study, patients were randomised to an intervention group (*n* = 40), where the DASH diet was introduced along with optimal pharmacotherapy, and to a control group (*n* = 39), with optimal pharmacotherapy alone. In the DASH group, systematic dietary counselling was provided for the follow-up period. RANTES and CXCL4 levels were determined using ELISA. Results: In the DASH group, the RANTES level insignificantly reduced from 42.70 ± 21.1 ng/mL to 38.09 ± 18.5 ng/mL (*p* = 0.134), and the CXCL4 concentration significantly reduced from 12.38 ± 4.1 ng/mL to 8.36 ± 2.3 ng/mL (*p* = 0.0001). At the same time, an increase in the level of both chemokines was observed in the control group: RANTES from 34.69 ± 22.7 to 40.94 ± 20.0 ng/mL (*p* = 0.06) and CXCL4 from 10.98 ± 3.6 to 13.0 5± 4.8 ng/mL (*p* = 0.009). The difference between the changes in both groups was significant for both RANTES (*p* = 0.03) and CXCL4 (*p* = 0.00001). The RANTES/CXCL4 ratio reduced in the control group (from 3.52 ± 2.8 to 3.35 ± 2.8; *p* = 0.006), while in the DASH group, an increase was observed (from 3.54 ± 1.7 to 4.77 ± 2.4; *p* = 0.001). Conclusions: A 12-month-long intensive dietary intervention based on DASH diet guidelines as an addition to optimal pharmacotherapy causes changes in the levels of chemokines CXCL4 and RANTES and their mutual relationship in comparison to conventional treatment.

## 1. Introduction

Studies on the dietary approaches to stop hypertension (DASH) diet show the efficacy of this nutrition model in the prevention and treatment of arterial hypertension [1,2] and reduction in the risk of common cardiovascular diseases (CVDs), such as coronary artery disease (CAD), stroke and heart failure [3,4,5,6]. The DASH diet is characterised by a high amount of fruits, vegetables, whole grains and low-fat dairy products and low content of saturated fats, cholesterol, cereals with low fibre content (high glycaemic index) and sweets [7]. One of the potential mechanisms underlying a positive effect of the DASH diet on the cardiovascular system may be via modification of the inflammatory process [8,9]. Inflammation is the key pathophysiological mechanism of the initiation and progression of atherosclerosis [10]. Studies conducted so far point to platelets as a probable link between inflammation and clotting activation, which is involved in atherosclerotic plaque destabilisation. Activated blood platelets secrete numerous chemokines, including CXCL4, CCL5, CXCL12 and others, which initiate or promote local inflammatory processes at the sites of vascular damage [11]. RANTES (also known as CCL5) belongs to the family of CC chemokines and has strong chemoattractive activity for T lymphocytes and macrophages [12], and its role in the development of atherosclerosis has been confirmed in numerous studies [13,14]. It was reported that subjects with metabolic syndrome and no clinically manifested cardiovascular disease show activation of blood platelets and increased serum RANTES levels [15]. However, studies on the correlation between serum RANTES levels and the severity of CAD are inconclusive. An increased RANTES level was observed in patients with myocardial infarction, especially complicated by left ventricular dysfunction [16,17], while the level of this chemokine in stable CAD decreased [18]. In other studies, in contrast, low levels of RANTES correlated with more advanced coronary atherosclerosis and were an independent predictor of increased cardiovascular mortality [19].

CXCL4, another platelet-derived chemokine, may cooperate with RANTES in the development and destabilisation of atherosclerotic lesions. CXCL4 (also known as platelet factor 4) is abundantly released after platelet activation. CXCL4 foster absorption of oxidised low-density lipoprotein (LDL) from macrophages, fostering formation of foam cells and promoting development of the lipid core of atherosclerotic plaque [11]. CXCL4 increases chemotaxis using RANTES and vice versa by creating CXCL4–CCL5 heterodimers, which triggers monocyte recruitment and adhesion [11,20]. Animal studies showed that injection of a peptide inhibitor of CXCL4-CCL5 heterodimer formation (MKEY), significantly reduced myocardial infarction size [21]. Nevertheless, similarly to RANTES, studies on the plasma CXCL4 level in patients with CAD have provided inconsistent results [22,23].

There is little research in the literature on the possibility of modifying the levels of chemokines RANTES and CXCL4 with dietary intervention. Considering that the co-presence of CXCL4 and RANTES increases monocyte recruitment to the endothelium more than CXCL4 or RANTES alone [24], documenting the effect on both chemokines seems pertinent. The current studies confirm the effect of Mediterranean diet variants on the reduction in RANTES concentration but do not explore its effect on the levels and proportions of CXCL4 [25].

The objective of the present study was to assess the effects of a dietary intervention based on the model of the DASH diet on the circulating levels of chemokines RANTES and CXCL4 in patients with known coronary atherosclerosis.

## 2. Materials and Methods

The study involved 97 participants of the Dietary Intervention to Stop Coronary Atherosclerosis in Computed Tomography (DISCO-CT) study, a pilot, single-centre, randomised study, who underwent coronary computed tomography angiography (CTA) as part of a routine diagnostics of CAD, which confirmed the presence of non-occlusive atherosclerotic lesions in at least two segments of the coronary arteries. All patients were carefully evaluated in terms of clinical status and supplementary tests and qualified to conservative treatment after no evidence of coronary artery obstruction had been found; subjects with clinical indications for expanded diagnostics of CAD and/or coronary revascularisation were not included. Patients with active neoplastic disease and diagnosed autoimmune disease were excluded from the study. The primary outcome of the trial was the reduction of percentage atheroma volume (PAV) measured between baseline and follow-up CT scans, whereas the secondary outcome was a change in pericardial fat distribution between baseline and follow-up CT scans. This article involves an analysis of additional data (serum inflammatory markers) regarding which research protocol was extended after 1 month upon commenced patient recruitment.

The inclusion criteria were as follows: coronary artery stenosis of <70% in at least two segments of the coronary arteries on CTA (according to the American College of Cardiology/American Heart Association classification) qualified for conservative treatment, age of >18 years, informed consent to take part in the study, declaration and willingness to cooperate and adherence to the doctor’s instructions. The following exclusion criteria were used: valvular heart disease requiring cardiac surgery (or expected intervention within 1 year), diagnosed dilated or hypertrophic cardiomyopathy, known genetic disorders affecting the development of atherosclerosis (e.g., familial hyperlipidaemia), factors that may affect the quality and safety of CTA, women of childbearing potential and insufficient quality of data obtained from CTA.

**Figure 1:**
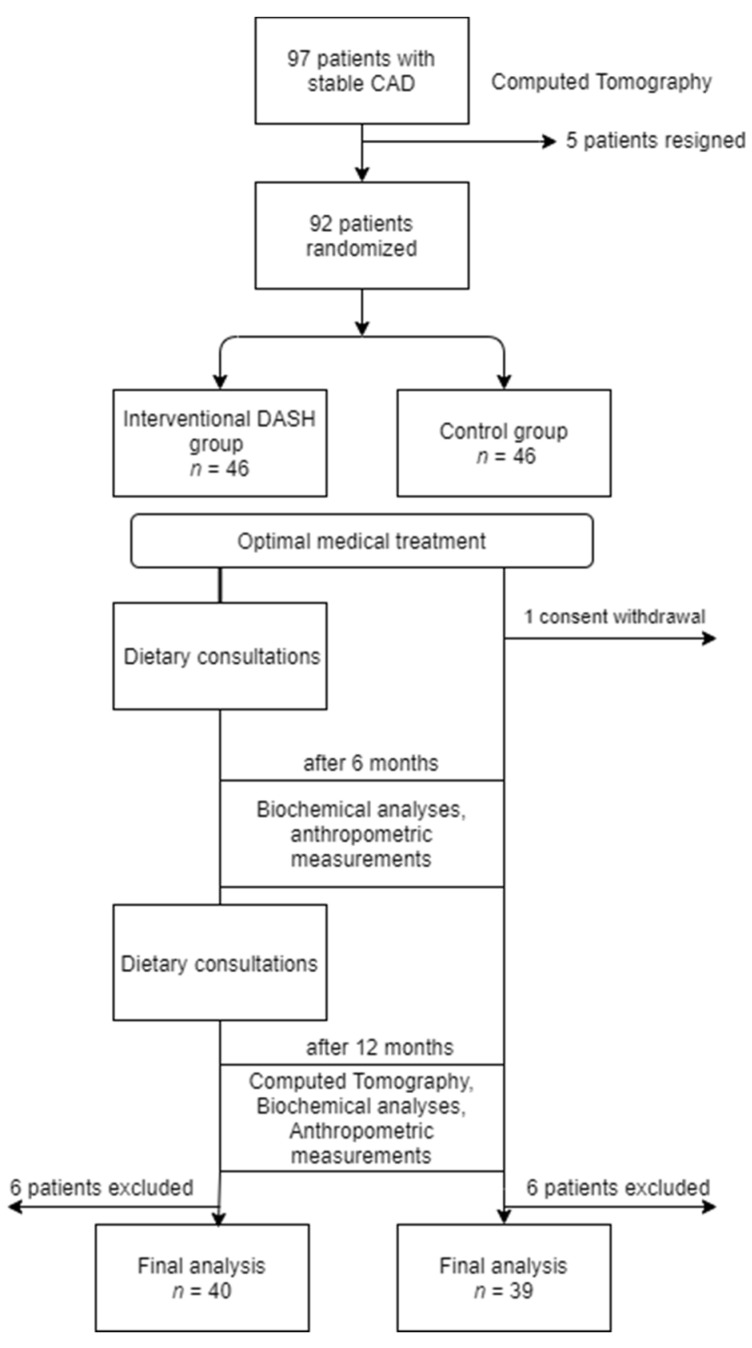
Flow diagram of participants’ allocation into study arms.

In this study, 92 subjects between 35 and 74 years of age (5 resigned during the course of the study) were randomly allocated to an experimental group with dietary intervention or to a control group, using 10 randomisation blocks with 10 subjects each at the ratio of 1:1. Patients from both groups received optimal medical treatment according to the European Society of Cardiology guidelines [26]. However, 1 subject withdrew consent, and 12 subjects were excluded from the final analysis (11 were diagnosed with rheumatoid arthritis, and 1 was excluded due to liver damage). The course of the study is shown in Figure 1.

### 2.1. Dietary Intervention

Dietary counselling involved the following visits: at the baseline and after 1, 3, 6, 9 and 12 months. Each subject was given the dietitian’s contact information (telephone number, e-mail address) so as to provide additional consultations. In the study group, the basal metabolic rate (BMR) was assessed on the basis of the patients’ body composition. In the intervention group, emphasis was placed on an increase in the number of meals to 5 and intervals between meals no longer than 3 h. On the basis of the BMR and the level of physical activity, the patients were assigned an individual DASH plans at the following caloric levels according to need: 1600, 1800, 2000 and 2600 kcal. The energy deficit in patients with obesity did not exceed 200 kcal a day and resulted from increased physical activity recommended for both groups. DASH diet plans supplied the following energy proportions at each energetic level: 52–55% from carbohydrates, 16–18% from proteins and 30% from total fats.

As part of the study, basic assessment of dietary habits was planned, including a survey of the meal frequency and a 24 h nutrition history. The assessment of diet quality and adherence was conducted in both groups with the use of the DASH index, according to the methodology described by Guntner et al. [27]. The DASH index comprises 8 food groups being part of the DASH diet: cereals, vegetables, fruits, dairy products, meat, nuts/seeds/pips, fats/oils and sweets. The maximum score in each food group can be 10.

### 2.2. Physical Activity

The patients in both groups were encouraged to perform increased physical activity throughout the study, and the instructions were compliant with the European Society of Cardiology guidelines [24]. At each follow-up visit, a history of physical activity during leisure time was taken. The patients were asked to classify their physical activity as regular (at least 3 sessions of 30 min per week), irregular or none.

### 2.3. Anthropometric Measurements

The patients’ height and body weight were measured at the baseline and after 6 months of intervention with the use of an electronic BSM370 device to the nearest 0.1 cm and 0.05 kg, respectively. The measurement of body composition was conducted twice, at the beginning of the study and after 12 months, with no shoes and minimum clothes using Body Water Analyzer InBody S10. The analysis included total body fat (TBF), total body fat percentage (TBF%) and visceral fat area (VFA) defined as the intra-abdominal cross-sectional area of visceral fat expressed in centimetres-squared. The measurements were conducted on an empty stomach, with at least a 12 h interval from physical activity.

### 2.4. Biochemical Analyses

Blood for biochemical tests was collected from fasting patients at the baseline visit, after 6 months and after 12 months. Plasma levels of CXCL4 and RANTES were assayed using ELISA (Human CXCL4 ELISA Kit; Biorbyt, Cambridge, UK, and Human RANTES ELISA Kit; Biorbyt, Cambridge, UK) according to the manufacturer’s protocol.

### 2.5. Calcium Score

The calcium score was assessed with dedicated software (syngo.via; Siemens, Erlangen, Germany) using non-contrast computed tomography scans and expressed in Agatston units.

### 2.6. Statistical Analysis

Statistical analysis of the results was performed using STATISTICA 13 software. Correlation was determined by calculating Spearman’s rank correlation coefficient. Means were compared using the *t*-test. Categorical data were compared using the Mann–Whitney *U* test.

The study was approved by the bioethics committee at the Institute of Cardiology in Warsaw (IK-NP-0021-51/1514/15). Written informed consent was obtained from each patient. The study protocol conformed to the ethical guidelines of the 1975 Declaration of Helsinki, as reflected in a priori approval by the institution’s human research committee. The trial was registered at clinicaltrials.gov (accessed on 7 October 2020) (NCT02571803).

## 3. Results

The characteristics of the study group and the control group are presented in Table 1.

During the 12 months of the study, all participants received the treatment according to the European Society of Cardiology guidelines [24]. The number of patients on statin treatment increased from 25 to 32 in the DASH group vs. from 29 to 33 in the control group. The study included 5 patients (6%) with documented statin intolerance and 9 patients (11%) who were unwilling to start pharmacotherapy of dyslipidaemia during the study. The DASH group revealed high adherence to the instructions of the DASH diet during the study, which resulted in significant improvement in the patients’ diet quality. The DASH index in the DASH group increased from 34.33 ± 14.7 to 60.47 ± 9.1 (*p* = 0.0001), while in the control group, such change was insignificant (increase from 34.56 ± 13.6 to 38.13 ± 12.3). Detailed changes in the specific components of the DASH index are presented in Table 2.

The number of patients declaring regular physical activity in the DASH group increased from an initial 22 (55%) to 32 (80%) (*p* = 0.04) vs. from 9 (23%) to 20 (51%) in the control group (*p* = 0.08). As a consequence of the intervention, we observed changes in the body composition. In the DASH group, a decrease in the total body fat percentage (TBF%) from 33.05 ± 9.1 to 30.10 ± 8.1 (*p* = 0.00015) was observed, while in the control group, there was a slight increase from 32.84 ± 7.34 to 33.51 ± 8.9 (*p* = 0.246). An important benefit of the dietary intervention was a reduction in the visceral fat content by 19.90 ± 29.2 cm^2^ in the DASH group (*p* = 0.0001), while in the control group the reduction was insignificant. In addition, a beneficial effect on the lipid profile was observed in the DASH group, with paired reductions by ca. 12% in total cholesterol and 16% in LDL, approximately twofold greater than in the control group.

### 3.1. Changes in Inflammatory Marker Levels

The analysis of selected demographic and clinical data by RANTES level quartiles and the coefficients of correlation with the RANTES level are presented in Table 3.

In the DASH group, no significant baseline correlation was observed between the RANTES level and the parameters of body composition and most of the analysed biochemical parameters, except for CXCL4 and homocysteine. For the high-sensitivity C-reactive protein (hsCRP), a significant correlation was observed between visceral fat (*r* = 0.252; *p* = 0.027) and the visceral fat percentage (*r* = 0.274; *p* = 0.024).

**Figure 2:**
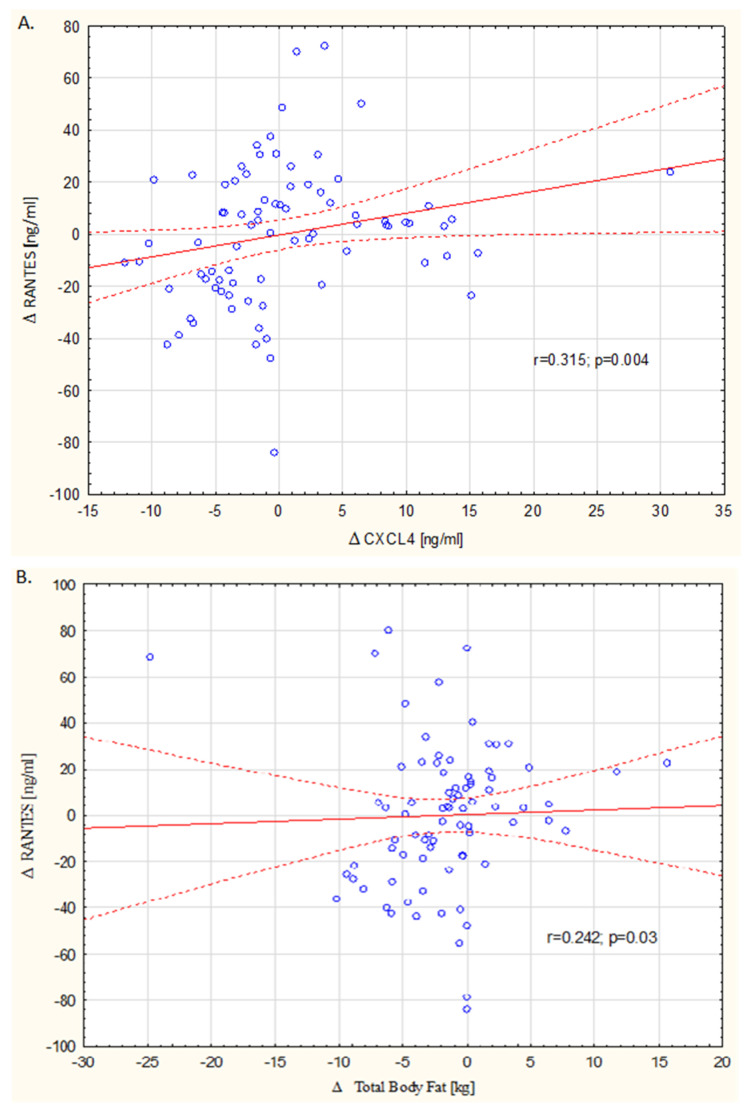
Correlation between a change in the RANTES level and a change in the CXCL4 level (**A**) and a change in total body fat (TBF) (**B**) during the study in all the patients.

During the course of the study, in the DASH group, a significant decrease in the hsCRP level from 0.23 ± 0.24 mg/L to 0.11 ± 0.13 mg/L (*p* = 0.003) was observed, while in the control group, there was an increase from 0.19 ± 0.17mg/L to 0.26 ± 0.75 mg/L (*p* = 0.275). A similar trend was observed in the level of both chemokines, although not in all cases were the changes statistically significant. The RANTES level insignificantly reduced from 42.70 ± 21.1 ng/mL to 38.09 ± 18.5 ng/mL (*p* = 0.134), and the CXCL4 level significantly reduced from 12.38 ± 4.1 ng/mL to 8.36 ± 2.3 ng/mL (*p* = 0.0001) in the DASH group. At the same time, an increase in the level of both chemokines was observed in the control group: RANTES from 34.69 ± 22.7 to 40.94 ± 20.0 ng/mL (*p* = 0.06) and CXCL4 from 10.98 ± 3.6 to 13.05 ± 4.8 ng/mL (*p* = 0.009). These results are presented in Table 4. Correlation analysis showed that for all the patients in both groups, a change in the RANTES level was correlated with a change in the CXCL4 level, which is shown in Figure 2A.

### 3.2. Correlation with DASH Index, Body Composition, Physical Activity and Calcium Score

Changes in the chemokine levels were significantly correlated with a change in the DASH index among all the patients (for CXCL4, r = −0.412, *p* = 0.003; for RANTES, r = −0.293, *p* = 0.04). In specific groups, they did not significantly correlate with a change in the total DASH index in any of the analysed groups. However, the DASH group revealed significant inverse correlations for a change in the CXCL4 level and a change in the index for vegetables (r = −0.310), the RANTES level for cereals (r = −0.363) and the RANTES/CXCL4 ratio for cereals, vegetables and fruits. Table 5 shows detailed results of the analysis regarding the correlation between changes in RANTES and CXCL4 levels in both groups and the DASH index, as well as its particular components.

Analysis of the correlation of the chemokine level change with the change in the total fat and visceral fat content showed a significant correlation only for RANTES and changes in the total fat content (*r* = 0.241; *p* = 0.03) in all the patients, but it was not confirmed in specific groups. The data scatter chart is shown in Figure 2B.

Correlation analysis did not show any relationship between physical activity and changes in the levels of both chemokines and their mutual relationship. Additionally, analysis of the correlation of chemokine levels with the calcium score was performed. After 12 months of study, the RANTES level was not significantly correlated with the calcium score (*r* = −0.135; *p* = 0.235), similar to the CXCL4 level (*r* = 0.059; *p* = 0.602). In addition, a change in both chemokines was not correlated with a change in the calcium score (for RANTES, *r* = −0.181, *p* = 0.107; for CXCL4, *r* = −0.02, *p* = 0.836).

## 4. Discussion

To the best of our knowledge, this is the first study showing the effect of an intensive dietary intervention based on the DASH diet on serial plasma levels of RANTES and CXCL4 in a long-term observation of patients with CAD. Reduced inflammation expressed as a decreased concentration of hsCRP and other inflammatory markers, such as interleukin 6 (IL-6), resulting from the use of the DASH diet has been confirmed by many previous studies. None of them, however, involved RANTES and CXCL4, and the follow-up period was usually 3 to 24 weeks [8]. Our observations show that a declining trend in inflammatory markers can be maintained beyond 24 weeks. Secondly, none of the up-to-date studies have aimed to investigate changes in chemokine levels over time. Last but not least, our study proves that the DASH diet is a good target nutritional model, since it may be used for a long time with satisfactory adherence.

Even though there is no doubt that RANTES and CXCL4 are important in the process of the formation and destabilisation of atherosclerotic plaque, much controversy around the predictive value of the discussed chemokines has been raised. In some studies, low RANTES levels (<19 ng/mL) were observed in healthy subjects, for example in a study by Parissis et al. [16] and Nomura et al., [15]. Other authors, however, indicate that low RANTES levels may predict negative cardiovascular events. Specifically, the literature on the clinical significance of the chemokine concentration in patients with CAD is inconsistent. For example, a study by Cavusoglu et al. demonstrated a significant increase in cardiovascular mortality in subjects who underwent coronary angiography with RANTES values in the lowest third (i.e., <19.49 ng/mL) [19]. In a study by Erbel et al., CXCL4 concentrations did not differ among patients with coronary artery disease and healthy controls [23]. Numerous studies have indicated that increased RANTES levels are associated with larger atherosclerotic lesions [28,29,30]. Moreover, in the Viriani study, an increased RANTES level was correlated with an increase in the total lipid-rich core volume but only in patients who did not use statins [29]. In a study by Versteylen et al., a higher level of RANTES predicted the presence of coronary stenosis of ≥50% assessed by coronary angiography [28]. Different results were obtained in the study by Podolec et al., which showed that the RANTES concentration was higher in subjects with less advanced atherosclerotic lesions (<50%) [31]. Such discrepancies may stem from differences in the study populations, methods of chemokine determination and assessment of atherosclerotic lesions. Additionally, Koper-Lenkiewicz et al. point to numerous factors that may affect the RANTES concentration, such as age, sex, platelet activation and race. They also hypothesise that serum RANTES levels are biomarkers of atheromatous lesion presence rather than markers of its severity [32]. Nevertheless, none of the previous studies focused on serial observation of chemokines over time, which is a novelty of our investigation.

We observed a significant decrease in the CXCL4 level in the DASH group by as much as 32% in the last 6 months of the observation (to 8.36 ng/mL). This seems to be a positive effect, considering the fact that in other studies, the CXCL4 level was in the range of 4.3 ng/mL to 8.7 ng/mL in healthy subjects and 5.8 ng/mL to 16.0 ng/mL in patients with CAD [22,33]. Contrarily, in the control group, there was a significant increase in the CXCL4 level by 18.8% in the last 6 months of the observation. As reported previously, the increase in the CXCL4 level in the first half of the study in the control group was only insignificant [34].

As for RANTES levels, we did not observe significant changes in any group, but the interchanges difference reached statistical significance (*p* = 0.03), which is worth noting. Baseline RANTES levels showed a significant inverse correlation with homocysteine and a positive correlation with the CXCL4 level. Moreover, changes in the RANTES concentration correlated with changes in the CXCL4 concentration, and changes in the RANTES/CXCL4 ratio strikingly differed in both groups. As previously mentioned, platelet CXCL4 forms heteromers with RANTES, which results in increased adhesion of monocytes to endothelial cells, while disturbing this interaction inhibits the formation of atherosclerotic plaques in hyperlipidaemic mice [35]. It seems, therefore, that a significant change in the ratio of both chemokines in the DASH group may have a beneficial effect with respect to atherosclerosis progression and destabilisation. This is also confirmed by our analysis of CTA results, in which a decrease in the vulnerable plaque component volume was twofold greater in the DASH group than in the control group [36].

More pronounced differences between the analysed groups observed for CXCL4 compared with RANTES may result from the fact that according to the current state of knowledge, statins modify RANTES concentrations but have no significant effect on CXCL4 levels [37,38], and in the present study, most patients were treated with statins (32 subjects in each group). CXCL4 might have been less prone to the impact of concomitant medication, as the only drug relevantly affecting its levels is warfarin (used by only one patient in the control group); acethylsalycic acid, commonly used in both groups, is not considered a confounding factor [39].

The study of the relationship between changes in the RANTES concentration and DASH diet elements revealed only a correlation with the consumption of whole grains in the DASH group. The mechanism of this action is not known. It is suspected that it may stem from the increased intake of magnesium, which is abundant in whole-grain products. An inverse correlation between dietary Mg intake and biomarkers of systemic inflammation, including hsCRP, interleukin-6 (IL-6) and tumor necrosis factor α (TNF-α), has been repeatedly confirmed both in cross-sectional and in prospective cohort studies on various populations [40]. This activity may also be attributed to high contents of dietary fibre, which may reduce the inflammatory process by slowing down the absorption of glucose, changing the intestinal microflora and stimulating the production of short-chain fatty acids [8].

A change in the CXCL4 level was significantly correlated with other components of the DASH diet, rather than with the RANTES level, i.e., with a change in vegetable consumption. The correlation of the change in the RANTES/CXCL4 ratio was significant for the consumption of vegetables, fruits and whole grains. Fruits and vegetables, aside to their dietary fibre content, may also reveal anti-inflammatory properties, thanks to specific antioxidant compounds [41,42]. However, there have been no literature reports on studies concerning the effects of consumption of particular nutrients on the levels of the analysed chemokines.

The change in the RANTES concentration in all the subjects was significantly correlated with a change in the total fat content, but it was not correlated with changes in hsCRP nor visceral fat. The analysis of specific groups revealed no significant correlation. It has been confirmed that RANTES is released by human subcutaneous adipose tissue in vivo, and RANTES concentrations in slim subjects (median 14.5 ng/mL) are lower than in obese subjects (median 29.4 ng/mL) and subjects with morbid obesity (median 57.0 ng/mL), but their share in the total concentration of circulating RANTES produced by various types of adipose tissue deposits is not decisive. According to the authors, ex vivo secretion of RANTES was higher from fat collected from the visceral area (gastric bulb) than from subcutaneous tissue (*p* = 0.001) [43]. Numerous studies have confirmed that excessive deposit of visceral fat is related with an increased risk of CAD [44]. One of the mechanisms of such action is probably the accumulation of leptin and the reduced number of its receptors, which leads to the production of proinflammatory cytokines (e.g., TNF-α and IL-1) and blocks the production of anti-inflammatory cytokines (e.g., IL-4) [45]. So far, there have been no detailed studies on the effect of a change in the visceral fat deposit on plasma RANTES concentrations. Therefore, it is difficult to assess to what extent the declining trend observed in the DASH group results from a decrease in the visceral fat deposit.

## 5. Conclusions

It may be concluded that a 12-month-long intensive dietary intervention based on the DASH diet guidelines, as an addition to pharmacotherapy causes, changes the levels of chemokines CXCL4 and RANTES and their mutual relationship in comparison to patients receiving conventional treatment. To confirm these results and for a more detailed explanation of the mechanism, it is necessary to conduct further studies on larger groups with controlled physical activity. 

## 6. Study Limitations

Patients treated with statins were not excluded from the study, since this pharmacotherapy is standard CAD treatment.

The study was not conducted as a cross-over study due to the long follow-up aiming to create a permanent change in patients’ lifestyle habits.

The intervention related to physical activity was not fully objectified in this study. All the patients were given the same instructions, but our analysis was based on the patients’ declarations provided during the interview concerning compliance with the instructions.

Since it was a pilot study, the sample size was quite small.

## Figures and Tables

**Table 1:** Characteristics of the study groups and concomitant medication at baseline and follow-up.

Clinical Data	Baseline			Follow-Up		
	DASH Group (*n* = 40)	Control Group (*n* = 39)	*p*	DASH Group (*n* = 40)	Control Group (*n* = 39)	*p*
Age (years)	59.3 ± 8.14	60.5 ± 7.81	0.226			
Sex: men (%)	27 (67)	20 (51)	0.261			
Observation time (weeks)	69.4 ± 15.8	64.3 ± 10.7	0.08			
Hypercholesterolaemia, *n* (%)	40 (100)	38 (97)	0.159			
Hypertension, *n* (%)	38 (95)	33 (85)	0.50			
Impaired glucose tolerance, *n* (%)	3 (7.5)	3 (8)	0.726			
Persistent atrial fibrillation, *n* (%)	3 (7.5)	3 (8)	0.938			
Chronic kidney disease, *n* (%)	0 (0)	1 (2.5)	0.851			
Current smoking, *n* (%)	4 (10)	9 (23)	0.245			
Prior smoking, *n* (%)	25 (62)	26 (67)	0.995			
Weight (kg)	84.4 ± 16.8	83.0 ± 15.56	0.330	81.6 ± 14.5	81.6 ± 15.9	0.487
Body mass index (kg/m^2^)	29.6 ± 4.15	29.09 ± 3.84	0.250	28.6 ± 3.6	28.6 ± 3.9	0.431
Maximal lumen diameter stenosis, %	36.6 ± 11.90	37.7 ± 10.30	0.413	32.2 ± 10.3	39.0 ± 12.4	**0.011**
Number of lesions >50%	2 (5)	2 (5)	>0.999	2 (5)	6 (15)	0.150
Systolic BP, mmHg	129.4 ± 11.97	129.4 ± 14.78	0.498	125.7 ± 13.6	128.2 ± 13.60	0.214
Diastolic BP, mmHg	79.7 ± 6.58	80.3 ± 8.66	0.368	76.8 ± 9.30	78.23 ± 5.61	0.222
**Treatment**						
β-Blocker, *n* (%)	21 (52.5)	26 (67)	0.115	22 (55)	27 (69)	0.338
ACE inhibitor, ARB *n* (%)	26 (65)	30 (77)	0.335	24 (60)	29 (74)	0.250
Calcium channel blocker, *n* (%)	13 (32)	10 (25)	0.703	12 (30)	11 (28)	0.703
Diuretic, *n* (%)	10 (25)	16 (41)	0.250	10 (25)	17 (43)	0.179
Number of antihypertensive drugs	1.8 ± 1.10	2.2 ± 1.31	0.090	1.7 ± 1.31	2.4 ± 1.28	**0.046**
Statins, *n* (%)	25 (62)	29 (74)	0.233	32 (80)	33 (84)	0.851
High-intensity-dose statins#, *n* (%)	7 (17.5)	7 (18)	0.996	9 (22.5)	8 (20.5)	0.851
Other lipid-lowering drugs, *n* (%)	3 (7.5)	2 (5)	0.859	2 (5)	1 (2.5)	0.856
ASA, *n* (%)	23 (57.5)	24 (61)	0.505	26 (65)	29 (74)	0.566
NOAC, *n* (%)	0 (0)	1 (2.5)	0.851	0 (0)	1 (2.5)	0.850
Warfarin, *n* (%)	0 (0)	1 (2.5)	0.851	0 (0)	1 (2.5)	0.851
Clopidogrel, *n* (%)	5 (12.5)	0 (0)	0.338	4 (10)	0 (0)	0.444

DASH, dietary approaches to stop hypertension; BP, blood pressure; ARB, angiotensin receptor blocker; ACE, angiotensin-converting enzyme; ASA, acetylsalicylic acid; NOAC, new oral anticoagulants; #atorvastatin 40 mg daily or more, rosuvastatin 20 mg daily or more. The *p* < 0.05 marked in bold.

**Table 2:** Baseline and final values of the DASH index and its components in both analysed groups.

Study Groups		Grains	Vegetables	Fruits	Dairy	Meat	Nuts	Fat	Sweets	DASH Index	*p*
**Control group**	Baseline	5.30	4.65	4.18	5.75	4.53	0.95	5.90	3.40	34.65	*p* = 0.138
	Follow-up	5.45	5.15	4.43	6.23	5.18	1.75	6.23	3.73	38.13	
**DASH group**	Baseline	5.35	4.65	4.15	7.60	4.38	0.80	5.85	3.30	34.33	*p* = 0.0001
	Follow-up	8.25	7.23	6.45	7.60	8.85	5.40	9.10	7.60	60.47	

**Table 3:** Demographic and selected baseline clinical data of all patients, divided by RANTES quartiles. Correlation coefficients of these data with baseline plasma RANTES levels.

Clinical and Laboratory Data	1st Quartile *n =* 19	2nd Quartile *n =* 21	3rd Quartile *n =* 19	4th Quartile *n =* 20	Correlation Coefficient with RANTES Levels	*p*-Value
CAD risk factors						
Age (years)	60.6	61.7	57.9	59.2	−0.179	0.110
Sex: men (%)	8 (42%)	12 (57%)	12 (63%)	16 (80%)	−0.192	0.087
Obesity (body mass index (BMI) >30), n (%)	8 (42%)	7 (33%)	13 (68%)	14 (70%)	0.156	0.165
Total body fat %	34.2 ± 8.5	30.5 ± 7.0	34.6 ± 7.7	32.5 ± 9.2	−0.034	0.780
Visceral fat (cm^2^)	110.00 ± 56.7	96.2 ± 35.1	111.5 ± 35.6	110.79 ± 40.1	0.088	0.434
Hypercholesterolaemia n, (%)	19 (100%)	20 (95%)	19 (100%)	19 (95%)	−0.053	0.635
Hypertension, n (%)	16 (84%)	19 (90%)	16 (84%)	19 (95%)	0.121	0.281
Calcium score (Agatson units)	144.81 ± 147.0	223.75 ± 217.1	118.5 ± 122.5	254.63 ± 125.2	0.037	0.746
Smoking (current and prior), n (%)	10 (52%)	10 (47.6%)	11 (58%)	16 (80%)	0.192	0.086
Laboratory data						
RANTES (ng/mL)	14.31 ± 5.6	27.26 ± 5.6	48.14 ± 5.0	68.75 ± 16.0	-	-
hsCRP (mg/L)	0.19 ± 0.19	0.21 ± 0.17	0.28 ± 0.32	0.18 ± 0.15	0.054	0.634
CXCL4 (ng/mL)	9.60 ± 3.3	11.69 ± 4.0	12.13 ± 3.7	12.95 ± 3.9	0.296	**0.007**
ALT (IU/L)	27.53 ± 15.2	26.33 ± 18.6	34.16 ± 17.9	28.10 ± 9.9	0.146	0.196
GGTP (IU/L)	40.21 ± 48.4	37.50 ± 41.6	34.16 ± 33.9	30.21 ± 15.2	0.097	0.398
TC (mg/dL)	199.08 ± 61.1	169.69 ± 36.1	171.35 ± 30.9	183.55 ± 40.1	−0.006	0.950
HDL (mg/dL)	61.35 ± 20.1	59.34 ± 13.9	55.07 ± 13.1	53.16 ± 11.2	−0.165	0.141
LDL (mg/dL)	121.43 ± 53.8	101.67 ± 31.6	101.16 ± 29.9	110.18 ± 30.5	−0.014	0.899
Homocysteine (µmol/L)	15.57 ± 12.2	14.35 ± 4.8	12.37 ± 3.0	11.66 ± 3.3	−0.247	**0.027**

hs-CRP, high-sensitivity C-reactive protein; GGTP, gamma-glutamyltransferase; ALT, alanine aminotransferase; TC, total cholesterol; LDL, low density lipoprotein; HDL, high-density lipoprotein; The *p* < 0.05 marked in bold.

**Table 4:** Changes in lipid profile, homocysteine and inflammatory marker levels during the study.

Biochemical Parameters	Study Groups	Baseline	6 Months Follow-Up	12 Months Follow-Up	Change (Δ)	*p*-Value	*p* * Value
Total cholesterol, mg/dL	DASH group	184.48 ± 44.8	169.43 ± 37.8	162.02 ± 35.1	22.46 ± 35.6	**0.007**	0.096
	Control	175.07 ± 43.8	169.11 ± 36.8	164.54 ± 32.4	10.53 ± 44.7	0.115	
LDL, mg/dL	DASH group	110.30 ± 36.3	98.54 ± 34.3	92.40 ± 29.1	17.89 ± 33.6	**0.008**	0.144
	Control	106.83 ± 40.6	100.57 ± 34.1	98.08 ± 27.1	−8.76 ± 42.0	0.133	
HDL, mg/dL	DASH group	56.31 ± 15.4	60.58 ± 15.7	59.78 ± 14.5	3.46 ± 7.6	0.151	0.158
	Control	58.48 ± 14.2	59.98 ± 15.8	60.17 ± 15.8	1.69 ± 8.1	0.311	
Triglycerides^,^ mg/dL	DASH group	73.21 ± 49.4	83.19 ± 50.25	95.06 ± 46.4	21.84 ± 50.8	**0.022**	**0.023**
	Control	111.83 ± 66.5	111.8 ± 50.06	102.71 ± 41.8	−9.12 ± 82.4	0.235	
hsCRP, mg/L	DASH group	0.23 ± 0.2	0.15 ± 0.15	0.11 ± 0.1	−0.12 ± 0.2	**0.003**	0.07
	Control	0.19 ± 0.8	0.17 ± 0.12	0.26 ± 0.8	0.07 ± 0.78	0.295	
Homocysteine, umol/L	DASH group	12.87 ± 4.2	12.05 ± 3.17	11.90 ± 4.0	−0.97 ± 2.6	0.147	0.364
	Control	14.04 ± 9.2	12.34 ± 2.8	12.20 ± 2.6	−1.84 ± 8.7	0.116	
RANTES, ng/mL	DASH group	42.70 ± 21.1	41.7 ± 20.7	38.09 ± 18.5	−4.66 ± 26.3	0.060	**0.030**
	Control	34.69 ± 22.7	32.72 ± 18.4	40.94 ± 20.0	6.25 ± 24.6	0.134	
CXCL4, ng/mL	DASH group	12.38 ± 4.1	9.27 ± 3.19	8.36 ± 2.3	−4.01 ± 3.1	**0.0001**	**0.00001**
	Control	10.98 ± 3.6	10.42 ± 4,9	13.05 ± 4.8	2.52 ± 5.0	**0.009**	
RANTES/CXCL4 ratio	DASH group	3,54 ± 1.7	4.75 ± 2.4	4.77 ± 2.4	1.23 ± 2.9	**0.001**	0.06
	Control	3.52 ± 2.8	4.20 ± 4.1	3.67 ± 2.8	0.15 ± 3.3	**0.006**	

*p* Indicates within-group differences (paired-samples *t*-test). *p* * Indicates differences between changes in both groups (paired-samples *t*-test). The *p* < 0.05 marked in bold.

**Table 5:** Correlation between the change in the RANTES, CXCL4 and RANTES/CXCL4 ratio and the change in the DASH index and its components.

Change in DASH Index	Study Groups	Δ CXCL4	Δ RANTES	ΔRANTES/CXCL4
ΔDASH index	DASH group	0.133; *p* = 0.410	−0.277; *p* = 0.08	−0.276; *p* = 0.08
	Control	0.167; *p* = 0.307	−0.06; *p* = 0.707	−0.213; *p* = 0.192
ΔGrains index	DASH group	0.044; *p* = 0.783	−0.363; ***p* = 0.02**	−0.344; ***p* = 0.02**
	Control	0.042; *p* = 0.796	0.128; *p* = 0.465	0.102; *p* = 534
ΔVegetable index	DASH group	−0.310; ***p* = 0.04**	−0.196; *p* = 0.22	−0.301; ***p* = 0.04**
	Control	−0.163; *p* = 0.320	−0.140; *p* = 0.394	−0.061; *p* = 711
ΔFruit index	DASH group	0.235; *p* = 0.142	−0.223; *p* = 0.164	−0.334; ***p* = 0.03**
	Control	0.146; *p* = 0.374	0.076; *p* = 0.654	0.011; *p* = 0.946
ΔDairy index	DASH group	0.09; *p* = 0.578	−0.051; *p* = 0.736	−0.01; *p* = 0.937
	Control	−0.170; *p* = 0.293	−0.183; *p* = 0.264	−0.102; *p* = 0.533
ΔNuts index	DASH group	−0.221; *p* = 0.893	−0.194; *p* = 0.230	−0.101; *p* = 0.534
	Control	0.116; *p* = 0.480	0.175; *p* = 0.286	0.007; *p* = 0.962
ΔFat index	DASH group	0.243; *p* = 0.130	−0.205; *p* = 0.202	−0.269; *p* = 0.09
	Control	0.001; *p* = 0.990	0.07; *p* = 0.653	0.003; *p* = 0.984
ΔMeat index	DASH group	0.230; *p* = 0.153	−0.128; *p* = 0.429	−0.16; *p* = 0.323
	Control	0.013; *p* = 0.932	−0.183; *p* = 0.264	−0.167; *p* = 0.307
ΔSweets index	DASH group	−0.001; *p* = 0.96	−0.228; *p* = 0.156	−0.210; *p* = 0.174
	Control	0.128; *p* = 0.434	−0.03; *p* = 0.816	−0.136; *p* = 0.408

The *p* < 0.05 marked in bold.

## Data Availability

The data presented in this study are available on request from the corresponding author. The data are not publicly available due to the study is funded by the Institute of Cardiology in Warsaw and management approval is required.
